# The evolution of sex-specific virulence in infectious diseases

**DOI:** 10.1038/ncomms13849

**Published:** 2016-12-13

**Authors:** Francisco Úbeda, Vincent A. A. Jansen

**Affiliations:** 1 School of Biological Sciences, Royal Holloway University of London, Egham, Surrey TW20 0EX, UK

## Abstract

Fatality rates of infectious diseases are often higher in men than women. Although this difference is often attributed to a stronger immune response in women, we show that differences in the transmission routes that the sexes provide can result in evolution favouring pathogens with sex-specific virulence. Because women can transmit pathogens during pregnancy, birth or breast-feeding, pathogens adapt, evolving lower virulence in women. This can resolve the long-standing puzzle on progression from Human T-cell Lymphotropic Virus Type 1 (HTLV-1) infection to lethal Adult T-cell Leukaemia (ATL); a progression that is more likely in Japanese men than women, while it is equally likely in Caribbean women and men. We argue that breastfeeding, being more prolonged in Japan than in the Caribbean, may have driven the difference in virulence between the two populations. Our finding signifies the importance of investigating the differences in genetic expression profile of pathogens in males and females.

## Abstract (web summary)

Many infectious diseases are more likely to progress to serious illness or death in men than in women, which has been attributed to a stronger immune response in women. Here, the authors propose that pathogen transmission from mother to child favours the evolution of lower virulence in women, and argue that the higher risk of HTLV-1 infection progressing to leukaemia in Japanese men is due to prolonged breastfeeding in Japan.

The progression and fatality rates (virulence) of many infectious diseases that do not affect sex-specific organs differ substantially between men and women^1^,^2^. For example, infection with the tubercle bacillus, *Mycobacterium tuberculosis* (MTB), may progress to active tuberculosis disease with high probability of causing death. Men infected with MTB are 1.5 times more likely to die than infected women are (shown in refs 3, 4, 5). Oral infection with Human Papilloma Virus (HPV) may progress to tonsil cancer, which is lethal. Men infected with HPV are five times more likely to develop cancer than women are (shown in refs 6, 7, 8, 9, 10, 11). Men are at least twice more likely than women to develop Hodgkin's lymphoma following infectious mononucleosis–related Epstein–Barr virus (EBV) infection^12^. Infection with Human T-cell Lymphotropic Virus Type 1 (HTLV-1) may progress to Adult T-cell Leukaemia (ATL) causing death. In Japan, men infected with HTLV-1 are between 2 and 3.5 times more likely to develop ATL and die than women are (shown in refs 13, 14, 15).

In spite of the mounting evidence suggesting that infectious diseases behave differently in each sex medical doctors tend to provide the same treatment to male and female patients^1^,^16^,^17^. One of the reasons why a sex-specific treatment has not been implemented is that the causes of sex-differences in virulence are not well understood^18^. The prevailing explanation posits that the interaction between sex-hormones and the immune system renders men more vulnerable to the action of the pathogen than women^1^,^16^. In general, females tend to mount a stronger immune response that helps them to clear infections faster and reduces the risk of persistence—although this response helps to fight infecting pathogens, it makes women more susceptible to immune pathologies^1^,^16^. While this hypothesis may account for some of the sex-differences observed, it does not provide a fully satisfactory answer; most prominently it does not explain why the sex-differences in virulence do not become apparent immediately after puberty—when sex-hormones are first produced—but almost a decade later^19^.

Seeking to improve our understanding of sex-differences in virulence, we move away from host-centred hypotheses—as previous explanations are—towards an alternative pathogen-centred hypothesis^20^. We propose that natural selection acting differently on pathogens in male and female hosts leads to pathogens exhibiting different virulence in men and women. There is some evidence for the existence of such sex-specific strategies: bacteria can have different strategies to exploit their hosts, depending on whether the host are male or female. For instance, several strictly maternally transmitted bacterial symbionts selectively kill male, but not female offspring^21^. *Pasteuria ramosa*, a parasite of *Daphnia magna*, can cause gigantism when in females, but not in males^22^. We will explore which factors lead to selection for differential exploitation strategies in pathogens residing in female and male hosts, and focus on the evolutionary implications of the different routes of transmission provided by each sex to the pathogens that they host. While both sexes are vectors of transmission to another individual in the same population (horizontal transmission), transmission from parent to offspring through pregnancy, birth or breast-feeding (maternal vertical transmission; henceforth vertical transmission) is limited to women.

Here we explore whether this fundamental biological difference alone can drive the evolution of sex-specific virulence in the pathogen.We advance evolutionary theory by formulating and solving analytically an extension of the classical susceptible-infected-recovered individuals (SIR) model^23^ that incorporates vertical and horizontal transmission between male and female hosts. We use this model to explore whether mixed transmission is expected to result in the evolution of sex-differences in virulence. We apply our results to explain the observed differences in virulence between men and women infected with HTLV-1 in two endemic regions: Japan and the Caribbean.

## Results

### Model formulation

**Figure 1:**
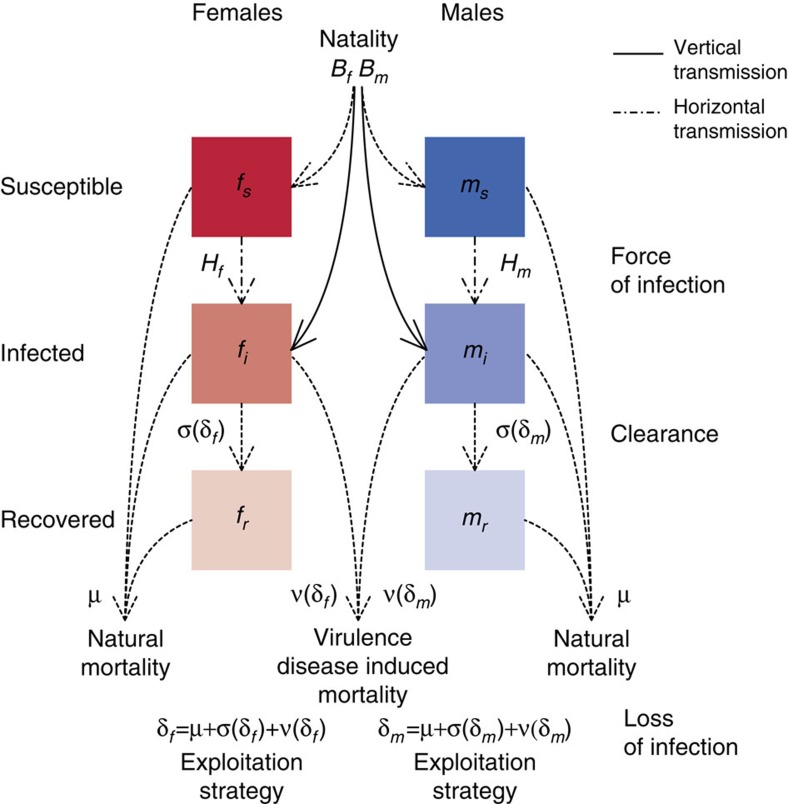
Diagram showing our epidemiological model of vertical and horizontal transmission in males and females. We model the continuous change in time of the fraction of susceptible (subscript *s*), infected (subscript *i*) and recovered (subscript *r*) individuals in males (*m*) and females (*f*). Males and females born, *B*_m_ and *B*_f_, are infected with probability *αf*_i_. Susceptible males and females, *m*_s_ and *f*_s_, become infected with probability given by the force of infection in males *H*_m_=*β(δ*_*m*_*)γ*_*mm*_*m*_*i*_*+β(δ*_*f*_*)γ*_*fm*_*f*_*i*_ and females *H*_f_=*β(δ*_*f*_*)γ*_*ff*_*f*_*i*_*+β(δ*_*m*_*)γ*_*mf*_*m*_*i*_. In a population at equilibrium every individual that dies is replaced with a new-born.

We extend the classical SIR model^23^ incorporating vertical and horizontal transmission between male and female hosts (see Fig. 1 and Methods for details). We solve this model allowing pathogens to exhibit different virulence in men and women.

There is a large literature on epidemiological models exploring the evolution of virulence in pathogens that are horizontally transmitted only^24^,^25^. There is a limited literature on epidemiological models exploring the evolution of virulence in pathogens that are vertically transmitted only^26^, showing that natural selection disfavours pathogens that are vertically transmitted only (when vertical transmission is uniparental) if they exhibit any degree of virulence^26^,^27^. Pathogens can only be maintained if they exhibit some special feature that compensates for the fitness loss caused by the death of their host^26^,^28^. Epidemiological models of pathogens that are only horizontally or vertically transmitted do not consider pathogens that may exhibit different virulence in men and women (but see ref. 29 for sex-specific virulence as a result of different resistance of male and female hosts).

There is also a large literature on behavioural models exploring the evolution of male killing by pathogens transmitted exclusively through cytoplasmic inheritance (male-killers)^30^,^31^, that is, equivalent to male limited virulence in pathogens that are vertically transmitted only. Natural selection does not disfavour male-killers because their virulence is limited to the sex that does not transmit them and thus does not translate in any fitness loss^32^. However, natural selection favours male-killers only when the death of a male translates into greater fitness of females closely related to the male-killer (*fitness compensation*)^28^.

Here we formulate an epidemiological model exploring the evolution of virulence in pathogens that are horizontally and vertically transmitted (*mixed transmission*). When there is mixed transmission, pathogens that are vertically transmitted can be maintained because they are also horizontally transmitted and not because they exhibit any form of fitness compensation^26^,^27^,^33^,^34^. Because of their complex nature, epidemiological models of mixed transmission are rare^27^,^33^,^34^,^35^,^36^, and they do not consider pathogens that may have different virulence in men and women. Thus, to the extent of our knowledge, ours is the first epidemiological model to consider pathogens with a sex-specific virulence. Most importantly, this model allows us to investigate the evolution of sex-specific virulence in pathogens causing infectious diseases—where horizontal transmission plays a role—when the only difference between the sexes is the presence of vertical transmission in women.

In our model, infection can take place either through vertical transmission at a rate α or horizontal transmission at a rate *β* per contact (Fig. 1). Horizontal transmission can either be from males or females, that is *β*_*m*_ and *β*_*f*_, where subscripts *m* and *f* denote males and females. Contacts can be established between any of the sexes, taking place at rates *γ*_*mm*_, *γ*_*mf*_, *γ*_*fm*_, and *γ*_*ff*_ with the first subscript indicating origin and the second destination. We assume that the number of contacts per unit of time is independent of the number of individuals in the population. An infected individual either recovers at a rate σ, dies from causes unrelated to the infection at a rate *μ* (natural mortality), or dies from causes related to the infection at a rate *ν* (virulence) (Fig. 1). Therefore, infections are lost from the population at a rate *δ* equal to the sum of these rates (*δ*=*σ+μ+ν*; Fig. 1). Another way of interpreting *δ* is as the reciprocal of the average duration of the infection: the higher the *δ*, the shorter the average duration of the infection will be. The rate of loss of infection *δ* is the pathogen's strategy to exploit its host (*exploitation strategy*). Crucially, we allow the pathogen's exploitation strategy to be different in men (*δ*_*m*_) and women (*δ*_*f*_) (Fig. 1).

### Trade-off between virulence and recovery and transmission

The higher the virulence *ν*, (and therefore the shorter the duration of the infection), the higher the horizontal transmission rate *β*^37^,^38^,^39^,^40^, that is *β*′(*δ*)>0 where prime denotes the derivative (see Supplementary Methods, Supplementary Table 1 for the notation used). We make the assumption—standard in evolutionary epidemiology models^24^,^25^,^41^—that there is a saturating trade-off between the horizontal transmission rate of a pathogen *β* from males and females, that is *β*_*m*_ and *β*_*f*_, and its virulence *ν* in males and females, that is *ν*_*m*_ and *ν*_*f*_, mediated by the sex-specific strategies *δ*_*m*_ and *δ*_*f*_ (refs 24, 25, 41), that is *β*_*m*_(*δ*_*m*_), *β*_*f*_(*δ*_*f*_) and *ν*_*m*_(*δ*_*m*_), *ν*_*f*_(*δ*_*f*_). Following classic vertical transmission models^33^,^34^ we make the assumption that the vertical transmission of a pathogen, α, is independent of its virulence, *ν*. Because we are interested in researching whether adding vertical transmission favours the pathogen to have different strategies in men and women (that is, whether evolution favours values of *δ*_*m*_ and *δ*_*f*_ that are not equal), we make the conservative assumption that the transmission-virulence trade-off is the same in pathogens residing in hosts of either sex, that is *β*_*m*_=*β*_*f*_=*β* and *ν*_*m*_=*ν*_*f*_=*ν*. Because transmission and virulence depend on the sex-specific exploitation strategies, this allows for the transmission and virulence of pathogens in women (*β*(*δ*_*f*_) and *ν*(*δ*_*f*_)) to be different from those residing in men (*β*(*δ*_*m*_) and *ν*(*δ*_*m*_)).

### ESS for a non-sex-specific strategy

We derive the fitness of a mutant pathogen and the exploitation strategy that once established in the population cannot be beaten by any alternative exploitation strategy (ESS)^32^,^42^. We do this by finding the exploitation for which the selection gradient is zero (see Methods section at the end of the paper and Supplementary Methods for details of the derivation). We show that the non-sex-specific (*δ*_*m*_=*δ*_*f*_=*δ*) ESS, 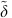, satisfies:


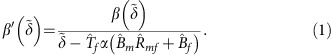


**Figure 2:**
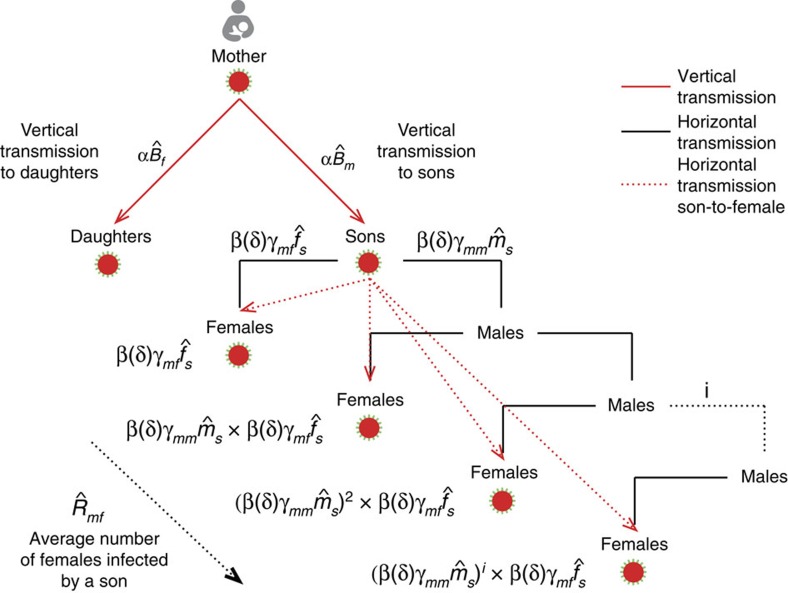
Interpretation of parameters. Interpretation of parameter 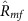 and expression 
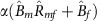
. 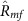 is the average number of females horizontally infected by a son and the male infections he will give rise to and results from adding up all possible transmission events to females. 
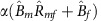
 is the effective rate of vertical transmission. It results from calculating the probability of vertical transmission to sons and daughters and horizontal transmission from son to females with whom he establishes contact.

This result renders itself to a simple interpretation: 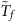 can be interpreted as the fraction of time a pathogen spends in females in a population at equilibrium, 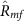 can be interpreted as the average number of females that an infected son infects through horizontal transmission at equilibrium, 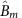 and 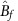 are the male and female birth rates at equilibrium (Fig. 2). The expression 
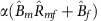
 can be interpreted as the effective rate of vertical transmission (Fig. 2). In the absence of vertical transmission, *α*=0, we recover the classic result for the evolution of virulence in pathogens with horizontal transmission only^24^,^25^.

### ESSs for sex-specific strategies

Solving for a different exploitation strategy in males and females, *δ*_*m*_ and *δ*_*f*_, we determine the ESS for a male-specific strategy, 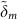:


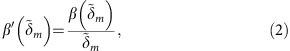


and the ESS for a female-specific strategy, 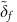:


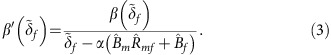


Notice that the ESS male-specific strategy 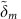 is the same as the ESS non-sex-specific strategy 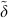 when virulence is not expressed in females, that is, when 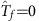, and the ESS female-specific strategy 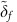 is the same as the ESS non-sex-specific strategy 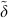 when virulence is only expressed in females, 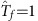. In the absence of vertical transmission, *α*=0, there is no difference in virulence between the male- and female-specific strategies. See Table 1 for a summary of results.

**Figure 3:**
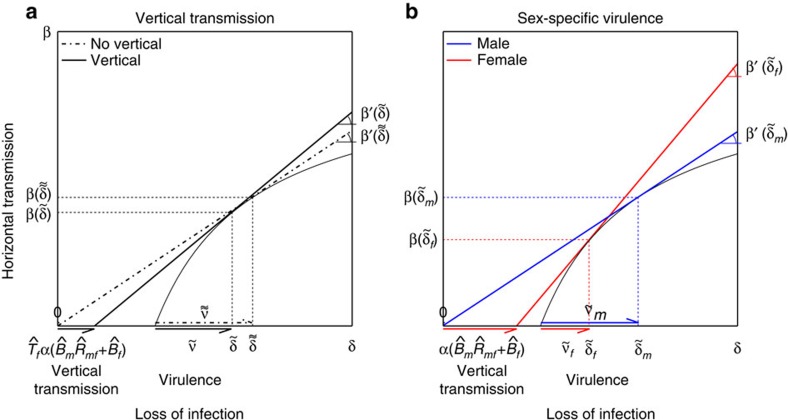
Interpreting the ESS virulence levels with a graphical method. We use a graphical method similar to the one developed by van Baalen and Sabelis^43^ to determine which ESS virulence will be greater. (**a**) Comparison between the ESS virulence with and without vertical transmission in the absence of sex-specific strategies. Without vertical transmission (*α*=0) the ESS exploitation strategy (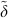) is the point where the tangent of the horizontal transmission function (*β*′(*δ*)) crosses the abscissa in *δ*=0. With vertical transmission (*α*>0), the ESS exploitation strategy (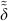) is the point where the tangent of the horizontal transmission function (*β*′(*δ*)) crosses the abscissa in 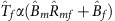. Notice that the tangent of the horizontal transmission function at the ESS is higher or equal with vertical transmission, that is 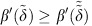. This implies that the ESS virulence is greater or equal without vertical transmission, that is 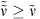. (**b**) Comparison between the ESS virulence when the virulence can differ in males and females. With male-specific virulence (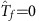) the ESS exploitation strategy (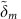) is the point where the tangent of the horizontal transmission function crosses the abscissa in *δ*_*m*_=0. With female-specific virulence (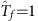) the ESS exploitation strategy (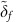) is the point where the tangent of the horizontal transmission function crosses the abscissa in 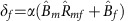. Notice that the tangent of the horizontal transmission function at the ESS is higher or equal with female-specific virulence, that is 
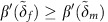
. This implies that the ESS virulence is greater or equal in males, 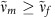.

**Figure 4:**
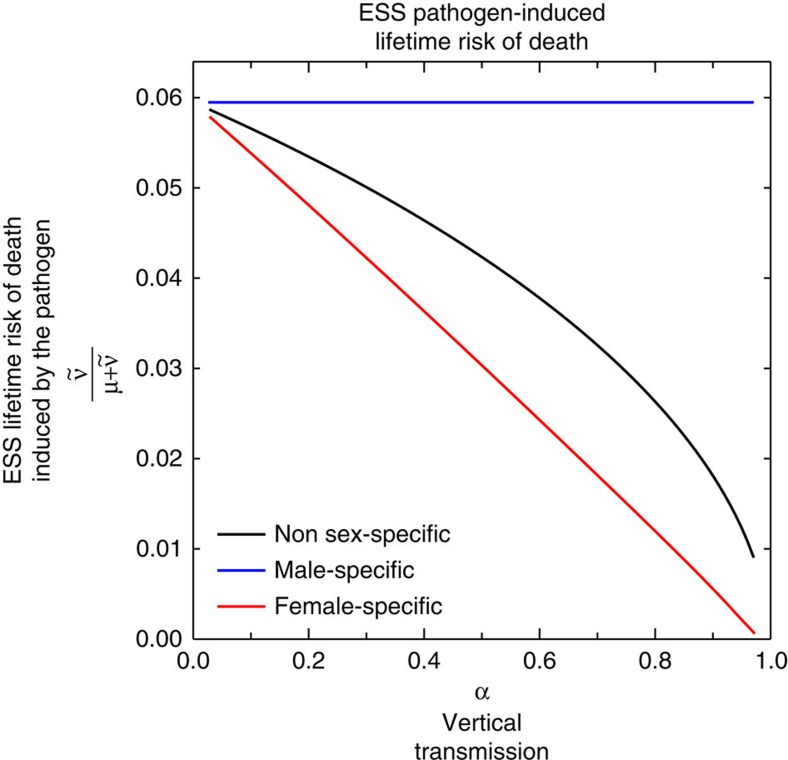
Evolutionarily stable virulence levels as a function of the rate of vertical transmission. The probability of dying of the disease over a lifetime (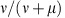) at the ESS for male specific, female specific and non-sex-specific exploitation strategies as a function of the rate of vertical transmission, *α* Parameters=1/80 *σ*_*f*_=*σ*_*m*_=0; *ϕ*=0.5, 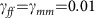; 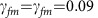; 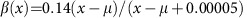 and 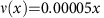. For these parameters the probability of dying of the disease for males, under male specific virulence will go to an ESS value of 6%.

Using a graphical method^43^ to analyse result (1) we confirm existing results indicating that a pathogen that is horizontally and vertically transmitted evolves to be less virulent than a pathogen that is horizontally transmitted only (Fig. 3a)^34^,^44^. Using the same graphical method to analyse results (2) and (3) we make two novel predictions: first, a pathogen that is horizontally and vertically transmitted is under selective pressure to evolve sex-specific virulence with lower virulence in females than males (Fig. 3b). The intuitive reason is that the existence of an additional route of transmission makes the life of hosts that transmit vertically—females but not males—more valuable to the pathogen. Second, the greater the vertical transmission the greater the difference in virulence between males and females a pathogen is selected for (Fig. 4). Therefore, given two populations that differ in their rates of vertical transmission, sex-specific virulence is more likely to be observed in the population with the greater rate of vertical transmission. Although this result is generic, the magnitude of the difference can depend on the model parameters (Supplementary Methods).

Evolutionary predictions are often hard to validate. However, in this case there is a unique natural experiment that allows us to test our prediction. HTLV-1 is a retrovirus that is horizontally transmitted via sexual intercourse and vertically transmitted via breast-feeding^13^. Infection with HTLV-1 can progress to ATL that is lethal. The Tax protein—encoded by the viral gene *tax*—orchestrates the oncogenic potential of the virus^45^,^46^, and therefore expression of the Tax protein is positively correlated to the virulence of the pathogen. The presence of anti-Tax antibodies is also an independent risk factor for sexual transmission^47^ but not for transmission through breast-feeding^48^. Therefore, the synthesis of Tax by HTLV-1 generates a trade-off between horizontal transmission and virulence in accordance with the assumptions of our model.

HTLV-1 is highly prevalent in two foci located in Southern Japan and the Caribbean^13^. These foci differ in the relative importance of each transmission route with breast-feeding being more important in Japan and sexual intercourse being more important in the Caribbean. This claim is sustained by three observations: (i) in Japan the spatial distribution of HTLV-1 has a patchy structure characteristic of vertical transmission^49^,^50^,^51^ while in the Caribbean the distribution is more uniform^52^,^53^; (ii) HTLV-1 transmission through breast-feeding (vertical transmission) is not sex-biased but transmission through sexual contact (horizontal transmission) is female-biased. In Japan prevalence of HTLV-1 is unbiased early in life and female biased later in life (from age 40-50) which is consistent with vertical transmission being more important there^50^,^51^,^54^ while in the Caribbean prevalence of HTLV-1 is female biased all through adult life (from age 20)^53^,^55^; (iii) Japanese women include breast-milk in their children's diet for longer time and in greater proportion than Caribbean women^56^,^57^,^58^ thus increasing the rate of vertical transmission per child^59^.

**Figure 5:**
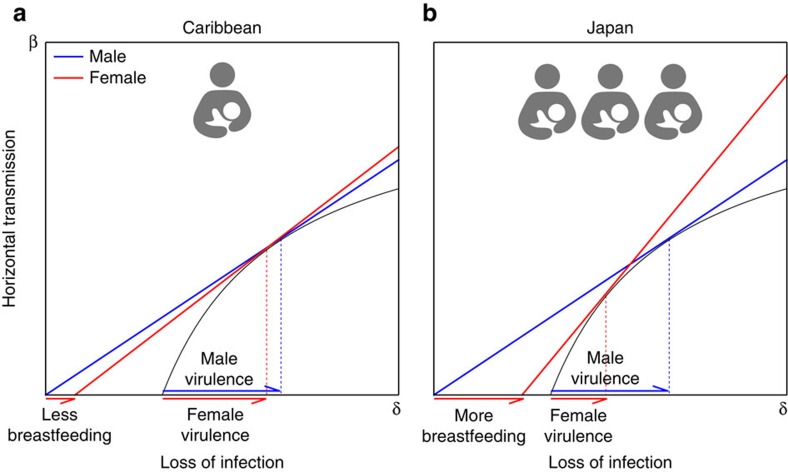
Predicted sex-specific virulence in two populations with different rates of vertical transmission. Predictions for the evolution of sex-specific virulence in two populations where the relative weight of vertical transmission is lower (**a**) (for example, the Caribbean where transmission through breast-feeding is low) and higher (**b**) (for example, Japan where transmission through breast-feeding is higher). Figures show how the difference in virulence between men and women is predicted to be greater in Japan than in the Caribbean.

**Figure 6:**
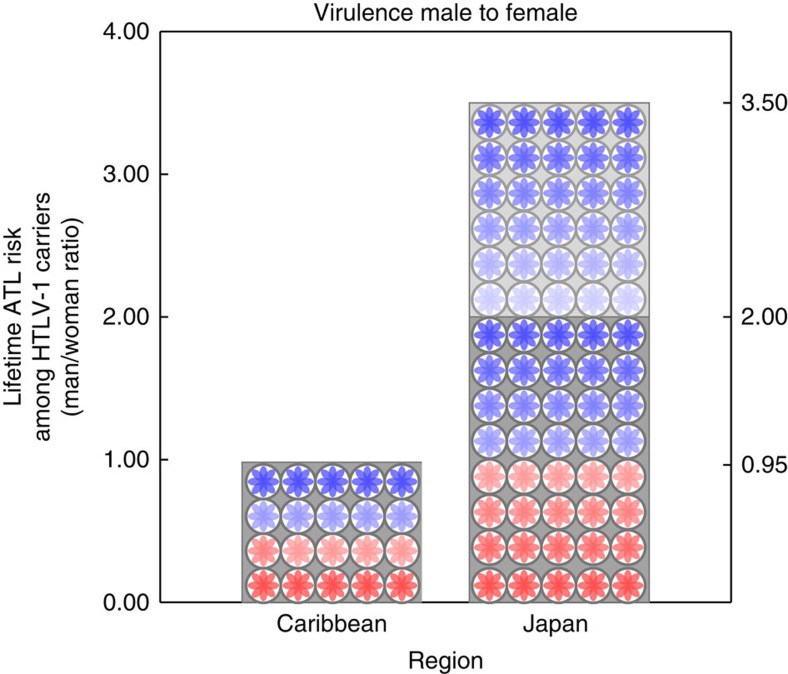
Sex-differences in the virulence of HTLV-1 in Japan and the Carribbean. Data on the lifetime risk of progression to ATL among male HTLV-1 carriers relative to female carriers. The lifetime risk of progression to ATL is approximately the same in men and women in the Caribbean^60^ but between 2.0 and 3.5 times more likely in men than women in Japan^14^.

That vertical transmission rate is larger in Japan than in the Caribbean leads us to predict that HTLV-1 should be less virulent in women relative to men in Japan than in the Caribbean (Fig. 5). This prediction is borne out by epidemiological data: Japanese men infected with HTLV-1 are between 2 and 3.5 times more likely to develop ATL than Japanese women are^13^,^14^,^45^. In contrast, Caribbean men infected with HTLV-1 are as likely to develop ATL as Caribbean women^13^,^52^,^60^ (Fig. 6). While it would be possible that this difference is caused by men being worse at fighting infectious diseases than women are in Japan but not in Jamaica it is unlikely. The male-to-female mortality ratio because of infectious diseases is 1.04 in Japan and 1.11 in Jamaica (GBD 2013 (ref. 61)). Notice that we would need the mortality ratio of those already infected in each country (which is not available) to provide a definitive answer but these figures suggest that the health of men relative to women in these countries do not differ significantly. There is also some indication that the virus acts differently between these two locations: significantly fewer Japanese carriers show an anti-Tax antibody response than Caribbean carriers do, which implies that the virus acts differently in the two regions and produces less Tax protein, and thus less leukaemia, in Japan^45^.

We suggest that the geographical differences regarding progression to ATL that have puzzled scientists in the last two decades^13^,^14^,^45^ are, at least in part, caused by a sex-specific adaptation of HTLV-1 virulence. In Japan, where the importance of breast-feeding transmission relative to sexual transmission is greater, natural selection on HTLV-1 favours slower progression to ATL in women than men thus preserving women as a viral route of transmission. However, in the Caribbean where the importance of breast-feeding relative to sexual transmission is lower, natural selection on HTLV-1 does not favour any difference in progression to ATL between women and men.

## Discussion

Our work shows that natural selection favours pathogens causing differential mortality in men and women when they are horizontally and vertically transmitted. In particular, pathogens are expected to evolve a degree of male virulence equal to that of pathogens in a population without vertical transmission and a degree of female virulence lower than that of pathogens in a population without vertical transmission (Fig. 3). Alternatively, pathogens are expected to evolve a degree of male virulence higher than that of pathogens in a population with mixed transmission but without sex-specific virulence and a degree of female virulence lower than that of pathogens in a population with mixed transmission but without sex-specific virulence (Figs 3 and 4). The intuitive reason why is that females, but not males, provide an additional route of transmission, making these hosts more valuable to the pathogen.

Our model predicts that pathogens that are transmitted horizontally and vertically through breastfeeding are more likely to evolve lower virulence in women in societies which breast-feed more or longer (greater rates of vertical transmission). This prediction matches the sex-specific virulence of HTLV-1 in Japan where vertical transmission is significant and the absence of differences in virulence in male and female hosts of the same virus in the Caribbean where vertical transmission is less important. Interestingly, this implies a social dilemma where extended breast-feeding practices may result, in the long run, in saving the life of women (who may experience a less virulent infection) by risking the lives of their children (who are more likely to acquire their mother's infection). A similar pattern is found in MTB, HPV and EBV which are all infections that can be transmitted from mother to child, and all these pathogens are significantly more virulent in males than in females. We hope this match between predictions and data will motivate the collection of more epidemiological data on sex-specific progression on other diseases that can ultimately validate the link between vertical transmission and sex-specific virulence.

Our work advances existing theory on the evolution of virulence by considering pathogens that can induce different mortality in male and female hosts. We provide a general analytic solution for the evolutionarily stable strategy of a pathogen that can be transmitted horizontally and vertically. This solution can accommodate any sex-specific contact structure and other sex-specific demographic parameters of their host. Furthermore, this solution can account for sex-specific (and non-sex-specific) virulence of the pathogen.

Our work puts the spotlight on pathogens responsible for infectious diseases as the potential cause of the observed difference in virulence between men and women. We model a fundamental biological difference between men and women—vertical transmission through breast-feeding, live birth or other means—and show that natural selection favours a departure in virulence between the sexes. Our work assumes no other biological difference between men and women, but in practice of course there are. Less fundamental differences, like sex-specific rates of horizontal transmission, sex-specific contact structures, may add to the difference in virulence between the sexes that can be attributed to the pathogen.

Our work questions the prevailing hypothesis that observed differences in virulence between the sexes have to be explained by differences in the reaction of the immune systems of men and women to an infection. On the debate of what causes the observed differences between the sexes (hormones, genes) we move away from hosts, where the debate is centred, to bring the pathogen's eye view. We argue that the cause can lie in the action of the pathogen and that the immune system of hosts alone will not provide a fully satisfactory answer—the action of pathogens alone will not either.

While most of the current medical research to understand sex-specific differences in virulence focuses on the expression profile of host genes^18^ our work invites to compare the expression profile of pathogen genes in pathogens infecting men and women. Current medical thinking implies manipulating the expression of host genes underpinning the immune reaction of men to provide an immune response in men similar to that in women. If evidence were to be found of different expression profiles of pathogen genes in pathogens infecting different sexes, medical research could attempt manipulating the cues used by pathogens to determine the sex of their host. By manipulating these cues, it may be possible to switch on the phenotype for women's virulence in pathogens infecting men thus reducing the mortality they induce.

A better understanding of what causes the observed virulence differences between men and women should bring us closer to implementing sex-specific medical treatment improving therapeutic choices.

## Methods

### The model

Here we develop a mathematical model that incorporates horizontal and vertical transmission between male and female hosts.

As we show in Supplementary Methods, a generic model for the change over time, *t*, in the fraction of susceptible and infected males, denoted as *m*_s_, *m*_i_, is:


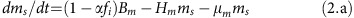



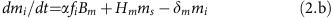


while the change in the fraction of susceptible and infected females, *f*_s_, *f*_i_, is:


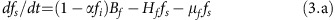



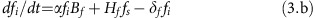


where *B*_*m*_ and *B*_*f*_ are the average birth rates of males per male (the total male birth rate divided by the number of males) and females per female and 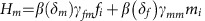 and 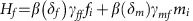 are the forces of infection on the male and female populations respectively. The fractions of recovered males and females, *m*_*r*_, *f*_*r*_ can be obtained from *m*_*s*_*+m*_*i*_*+m*_*r*_=1 and *f*_*s*_*+f*_*i*_*+f*_*r*_=1 respectively (see Supplementary Methods; Supplementary Table 1 for a list of parameters and variables used).

### Fitness

Next, consider the fate of a rare novel pathogen strain (which we will refer to as the mutant) entering a population with an established pathogen (which we will refer as the wild type) at equilibrium, with equilibrium values 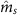 and 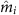 and females, 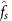 and 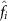. At equilibrium the number of births equals the number of deaths (both natural and caused by the disease) so that the total number of individuals in the population remains constant and we have the equilibrium birth rates 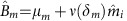 and 
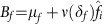
.

The mutant differs from the wild type in the parameters that govern host exploitation,: 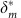, 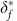. The linearized growth rate of the mutant's infected densities are governed by the matrix


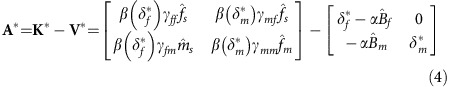


The fitness, *W*, of the mutant pathogen in a population dominated by the wild type is the dominant eigenvalue of the next generation matrix K^*^V^*−1^ (refs 62, 63), which makes *W* a function of the traits of mutant and wild type pathogens. The ESS is the exploitation strategy that maximizes the fitness of the mutant when the mutant plays the wild type's strategy. Therefore at the ESS the selection gradient of the mutant pathogen is equal to zero^64^.

### ESS calculation

To calculate the selection gradient we need the right and left eigenvectors of the matrix A*, associated with eigenvalue 0, when the mutant's strategy is the same as the wild type strategy (see Supplementary Methods for a more detailed derivation). The right eigenvector is 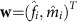 and the left eigenvector is 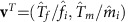, where the compound parameter


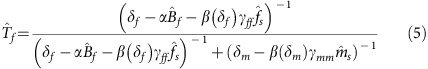


can be interpreted as the fraction of time the infection spends in females, and the parameter


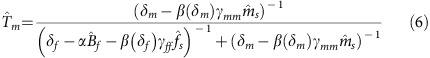


as the fraction of time the infection spends in males. Using this methodology we were able to find the ESS strategy in general.

We next calculate the ESS strategy in three specific cases: (1) when the mutant strategy has an effect on males only (male-specific), (2) when the mutant strategy has an effect on females only (female-specific) and (3) when the mutant strategy has the same effect in males and females (not sex-specific). We do this under the assumption that there are no inherent physiological differences between the sexes and that the trade offs work the same in both sexes, that is *μ*_*f*_=*μ*_*m*_=*μ*, 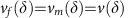, and 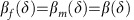.

### ESS for a strategy that is male host specific

If the mutant strategy is male-specific the ESS exploitation strategy 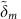 is given by:


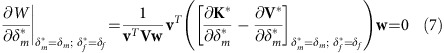


where 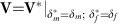.

This condition reduces to:


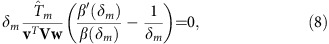


and the ESS exploitation strategy 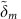 satisfies:


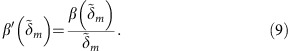


### ESS for a strategy that is female host specific

if the mutant strategy is female-specific the ESS exploitation strategy 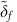 is given by:


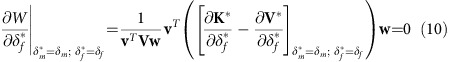


This condition reduces to:


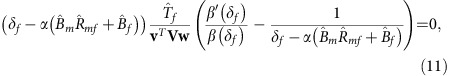


and the ESS exploitation strategy 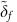 satisfies:


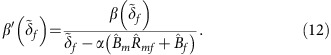


The compound parameter 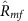 is:


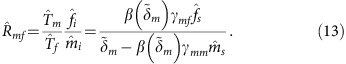


and can be interpreted as the average number of infected females that result from an infected son (Fig. 2). This includes all infections to a female in the male line of transmission, as can be seen by, adding up over all transmission events to a female that a pathogen in the male line can achieve:


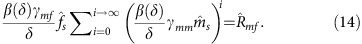


Notice that both 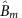 and 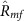 depends explicitly on 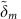 so that for female specific strategies the ESS value for *δ*_*f*_ depends on 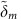.

### ESS for a strategy that is not sex-specific

If the mutant strategy is not sex-specific the ESS exploitation strategy 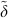 is given by:


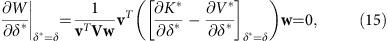


This condition reduces to:


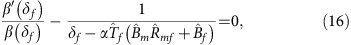


and the ESS exploitation strategy 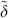 satisfies:


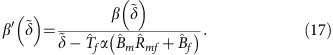


Notice that when the exploitation strategy is male-specific, parameter 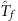 takes the value 0 and we recover the ESS result for 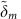. When it is female-specific, parameter 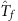 takes the value 1 and we recover the ESS for 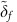. When the strategy is not sex-specific. parameter 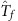 is comprised between 0 and 1.

More comprehensive results are shown in the Supplementary Methods. Our general theory makes it is easy to consider particular cases of pathogen transmission, for example the case of HTLV-1: horizontal transmission through heterosexual contact. When transmission is strictly through heterosexual contact *γ*_*mm*_*=γ*_*ff*_=0 and parameters 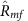 and 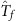 greatly simplify.

### Data availability

No data sets were generated during the current study. The data used in text or figures is in the public domain and were retrieved from the sources mentioned. For any further details, including computer code used, please contact rdm@royalholloway.ac.uk.

## Additional information

**How to cite this article:** Úbeda, F. & Jansen, V. A. A. The evolution of sex-specific virulence in infectious diseases. *Nat. Commun.* **7,** 13849 doi: 10.1038/ncomms13849 (2016).

**Publisher's note:** Springer Nature remains neutral with regard to jurisdictional claims in published maps and institutional affiliations.

## Supplementary Material

Supplementary Information Supplementary Table, Supplementary Methods and Supplementary References.

## Figures and Tables

**Table 1:** Summary of results on the ESS host exploitation condition.

**Transmission**	**Virulence**		
	**Not sex-specific**	**Sex-specific**	
		**In male hosts**	**In female hosts**
Horizontal *α*=0	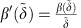	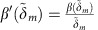	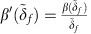
Horizontal & Vertical 0<*α*<1	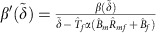	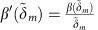	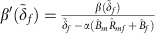

The table summarizes the specific results following from our main results on ESS conditions. Columns present the ESS virulence when virulence is not sex-specific, male-specific and female-specific. Rows present the ESS virulence when there is horizontal transmission only, and when there is horizontal and vertical transmission.
