# Supplementary material for: The evolution of sex-specific virulence in infectious diseases
Source: Nat Commun. 2016 Dec 13;7:13849. doi: 10.1038/ncomms13849 (PMC5159935; doi:10.1038/ncomms13849)
Supplement: Supplementary Information — Supplementary Table, Supplementary Methods and Supplementary References. [file ncomms13849-s1.pdf]

Supplementary Methods

Supplementary material to: Úbeda, F. & Jansen, V.A.A. (2016) The Evolution of Sex-specific Virulence in Infectious Diseases, published in *Nature Communications*

Supplementary Table 1

Notation used in this paper:

N	Number of individuals
S	Number of susceptible individuals
I	Number of infected individuals
M	Number of males
F	Number of females
M_s, M_i, M_r	Number of susceptible, infected, and resistant males
F_s, F_i, F_r	Number of susceptible, infected, and resistant females
m_s, m_i, m_r	Fraction of susceptible, infected, and resistant males
f_s, f_i, f_r	Fraction of susceptible, infected, and resistant females
α	Vertical transmission rate
μ_a	Mortality rate of class a
B	Birth rate
B_f	Average birth rate of females ($(1-\phi)B/F$)
B_m	Average birth rate of males ($\phi B/M$)
ϕ	Fraction of the population born as male
β_a	Horizontal transmission rate from class a
$\beta(x)$	Horizontal transmission rate as a function of trait x.
H_a	the force of infection experienced by class a.
γ_{ab}	Contact rate between individuals of classes a and b
δ_a	Rate with which infections are removed from class a
σ_a	Recovery rate in class a
ν_a	Virulence (disease related mortality rate) in class a
τ	Wild type trait
τ^*	Mutant trait
$\tilde{\tau}$	ESS trait value
$\hat{R}_{mf}$	Total number of infected females from an infected male
$\hat{T}_f$, $\hat{T}_m$	Time pathogens spend in females (males)
$\mathbf{A}^*$	Invasion matrix for a mutant
$\mathbf{A}$	Invasion matrix for mutant which has the same traits as the wild type
$\mathbf{w}$	Right eigenvector of $\mathbf{A}$, also vector that contains equilibrium fractions
$\mathbf{v}^T$	Left eigenvector of $\mathbf{A}$

Model Formulation

Here we present a general model for the vertical and horizontal transmission of a pathogen in a host population of males and females. We will calculate the fitness and evolutionarily stable strategies.

Let N be the total number of individuals in a population. Let F and M be the total number of females and males in that population, notice that $F + M = N$. Within each sex individuals might be either uninfected and thus susceptible to being infected with a pathogen (denoted by subscript s, that is F_s and M_s), infected (denoted by subscript i, that is F_i and M_i), or recovered from infection and thus not susceptible to being infected (denoted by subscript r, that is F_r and M_r), notice that $F = F_s + F_i + F_r$ and $M = M_s + M_i + M_r$.

The pathogen can be transmitted either vertically from mother to child at a rate α for each birth, or horizontally from infected individual of class a at a rate β_a per contact. Per unit of time an individual from class a encounters on average γ_{ab} individuals of class b. The subscripts $a, b \in \{f, m\}$ where f, m represent the classes males and females respectively. The total transmission per unit of time from individuals in class a to class b is thus $\beta_a \gamma_{ab}$ [1]. We assume that the number of contacts per unit of time is independent of the size of the classes within the population, or the size of the population. We also assume that the pathogen cannot alter the rates of contact.

Infections are removed from the population at rate $\delta_a = \sigma_a + \mu_a + \nu_a$, either through recovery at a rate σ_a, natural death at a rate μ_a, or death caused by the disease (virulence) at a rate ν_a. The average duration of the infection in an individual of class a is thus δ_a^{-1}. Generally it is assumed that the three parameters that describe the epidemiology of the pathogen, namely transmission, recovery and virulence, are genetically linked and that this creates a trade-off [2]. We assume therefore assume that these three rates be controlled by two pathogen traits, τ_a, with $a \in \{f, m\}$, one of which controls the trade-off in male hosts, and one in female hosts, if virulence is host-specific. If the virulence is not host specific we will set $\tau_f = \tau_m$. We can thus write $\delta_a(\tau_a) = \sigma_a(\tau_a) + \mu_a + \nu_a(\tau_a)$. Note that we have implicitly assumed that recovery trades off with virulence. This formulation in terms of traits τ_a allows us to parametrise the trade off in a generic manner. In the literature it is often assumed that recovery is independent of virulence; this is easily recovered in our model as a special case. This is easily recovered here by choosing $\sigma_a(\tau_a) = 0$ and $\nu_a(\tau_a) = \tau_a$.

We assume that the transmission from females and males, β_f and β_m respectively, depends on trait of the pathogen, and this can be specific towards the sex of the host that the pathogen is residing in. We will assume that transmission from female hosts depends on female specific traits of the pathogen, τ_f, and we can thus write $\beta_f(\tau_f)$ if we want to indicate the dependence of the transmission rate on the trait. We make a similar assumption for pathogens residing in male hosts so will write $\beta_m(\tau_m)$. The rationale for this assumption is that the transmission often depends on the pathogen load within a host. A higher load leads to more transmission, but also to an increase in the host mortality rate (virulence). As both are controlled by the same trait, there is a trade-off between the emission of infectious material and the virulence. As an increased load can also lead trigger a stronger immune response, this trait also controls the recovery rate [2]. When the transmission or recovery rate are not expressed as a function of a trait it is assumed that they depend on the trait values of the wild type τ_f and τ_m.

The birth rate into the population is given by B. The birth rate can be density dependent, it a function of the number of males and females and it can depend on the infection status of the individuals within the population, but we will not make this dependence explicit. The birth rate of males and females is given by ϕB and $(1-\phi)B$, respectively, so the that sex ratio at birth is $\frac{\phi}{1-\phi}$.

Let superscript dots refer to the derivative of a function respect to time t. The change over time in the total number of individuals in each sex and epidemiological class is given by:

$$\begin{array}{rcl} \dot{F}_s & = & (1-\phi)B\frac{F-\alpha F_i}{F} - \left(\beta_f\gamma_{ff}\frac{F_i}{F} + \beta_m\gamma_{mf}\frac{M_i}{M}\right)F_s - \mu_f F_s \\ \dot{F}_i & = & (1-\phi)B\frac{\alpha F_i}{F} + \left(\beta_f\gamma_{ff}\frac{F_i}{F} + \beta_m\gamma_{mf}\frac{M_i}{M}\right)F_s - \delta_f F_i \\ \dot{F}_r & = & \sigma_f F_i - \mu_f F_r \end{array} \quad (1)$$

in females, and

$$\begin{array}{rcl} \dot{M}_s & = & \phi B\frac{F-\alpha F_i}{F} - \left(\beta_f\gamma_{fm}\frac{F_i}{F} + \beta_m\gamma_{mm}\frac{M_i}{M}\right)M_s - \mu_m M_s \\ \dot{M}_i & = & \phi B\frac{\alpha F_i}{F} + \left(\beta_f\gamma_{fm}\frac{F_i}{F} + \beta_m\gamma_{mm}\frac{M_i}{M}\right)M_s - \delta_m M_i \\ \dot{M}_r & = & \sigma_m M_i - \mu_m M_r \end{array} \quad (2)$$

in males.

Let the fraction of susceptible, infected, and recovered females be $f_s = \frac{F_s}{F}$, $f_i = \frac{F_i}{F}$, and $f_r = \frac{F_r}{F}$ respectively with $f_s + f_i + f_r = 1$. Similarly, let the fraction of susceptible, infected, and recovered males be $m_s = \frac{M_s}{M}$, $m_i = \frac{M_i}{M}$, and $m_r = \frac{M_r}{M}$ respectively with $m_s + m_i + m_r = 1$. It is now possible to write the system of equations (1) in relative terms where the fraction of recovered individuals

can be derived from the fraction of susceptible and infected. The dynamics are:

$$\begin{aligned} \dot{f}_s &= B_f(1-\alpha f_i) - (\beta_f \gamma_{ff} f_i + \beta_m \gamma_{mf} m_i) f_s - \mu_f f_s - f_s(B_f - \mu_f - \nu_f f_i) \\ \dot{f}_i &= B_f \alpha f_i + (\beta_f \gamma_{ff} f_i + \beta_m \gamma_{mf} m_i) f_s - \delta_f f_i - f_i(B_f - \mu_f - \nu_f f_i) \end{aligned} \tag{3}$$

in females, and

$$\begin{aligned} \dot{m}_s &= B_m(1-\alpha f_i) - (\beta_f \gamma_{fm} f_i + \beta_m \gamma_{mm} m_i) m_s - \mu_m m_s - m_s(B_m - \mu_m - \nu_m m_i) \\ \dot{m}_i &= B_m \alpha f_i + (\beta_f \gamma_{fm} f_i + \beta_m \gamma_{mm} m_i) m_s - \delta_m m_i - m_i(B_m - \mu_m - \nu_m m_i) \end{aligned} \tag{4}$$

in males. The total number of males and females M and F, change over time as

$$\begin{aligned} \dot{F} &= F\left(B_f - \mu_f - \nu_f f_i\right) \\ \dot{M} &= M\left(B_m - \mu_m - \nu_m m_i\right), \end{aligned} \tag{5}$$

where $B_f = (1-\phi)\frac{B}{F}$ is the birth of females per female and, likewise, $B_m = \phi\frac{B}{M}$ is the birth of males per male. In what follows we will need the equilibrium fractions of susceptible and infected males, $\hat{m}_s$ and $\hat{m}_i$ and females, $\hat{f}_s$ and $\hat{f}_i$, and the equilibrium birth rates at equilibrium $\hat{B}_f$ and $\hat{B}_m$. These can be found by setting the left hand sides of the (3), (4) and (5) to zero.[1] Note that it follows from setting equations (5) to zero that $\hat{B}_f = \mu_f + \nu_f \hat{f}_i$ and $\hat{B}_m = \mu_m + \nu_m \hat{m}_i$. Solving these equations in closed form to give transparent results is often not practically possible, other than in the simplest cases. It is for this reason probably that most studies in the literature resort to numerical techniques and simulations when studying systems of this form. Here we will derive results for the evolutionarily stable values of τ_m and τ_f, which are functions of the equilibrial values of the densities and birth rates. This allows us to gain insight into this dependence without having to calculate the equilibrial values explicitly. In cases where we do need to calculate specific values this can be easily done numerically.

Fitness

We consider a novel pathogen strain (which we refer to as a mutant) entering a population of an established pathogen (which we will refer to as the wild type) at a dynamical equilibrium between the different classes of individuals. The mutant differs from the wild type in its traits τ_f^* and τ_m^*. Henceforth, superscript star denotes the variables and parameters associated the mutant strain. We assume that the mutant and the pathogen are sufficiently similar so that there is complete cross immunity.

In what follows we will, following [3-5], do an invasion analysis if the mutant strain is rare. If that is the case, the resident dynamics will be approximately be given by the eqns (3-5). The dynamics of individuals infected by the mutant strain (which we will denote by F_i^* and M_i^* for females and males respectively) are given by

$$\begin{aligned} \dot{F}_i^* &= (1-\phi)(B+B^*)\frac{\alpha F_i^*}{F^*} + \left(\beta_f(\tau_f^*)\gamma_{ff}\frac{F_i^*}{F^*} + \beta_m(\tau_m^*)\gamma_{mf}\frac{M_i^*}{M^*}\right) F_s - \delta_f(\tau_f^*)F_i^* \\ \dot{M}_i^* &= \phi(B+B^*)\frac{\alpha F_i^*}{F^*} + \left(\beta_f(\tau_f^*)\gamma_{fm}\frac{F_i^*}{F^*} + \beta_m(\tau_m^*)\gamma_{mm}\frac{M_i^*}{M^*}\right) M_s - \delta_m(\tau_m^*)M_i^*. \end{aligned}$$

Here $F^* = F + F_i^*$, $M^* = M + M_i^*$, and B^* is the differential birth that is caused by the presence of the mutant pathogen strain. Even though we have not specified the birth rate and how it depends on the population size or infection status, it stands to reason that if the mutant pathogen is absent there is no effect on the birth rate, and that if the mutant pathogen is rare in the population, the additional birth rate will be proportional to the number of individuals that are, or have been, infected with the mutant pathogen.

[1] It is also possible to deal with the case where the fractions go to equilibrium but the total population does not and grows exponentially.

We will next describe the fractions of males and females infected with the mutant strain. If the mutant strain is rare, the dynamics of the infected males and females are given, to first order, by:

$$
\begin{aligned}
\dot{F}_i^* &= (1-\phi)B\frac{\alpha F_i^*}{F} + \left(\beta_f(\tau_f^*)\gamma_{ff}\frac{F_i^*}{F} + \beta_m(\tau_m^*)\gamma_{mf}\frac{M_i^*}{M}\right)F_s - \delta_f(\tau_f^*)F_i^* \\
\dot{M}_i^* &= \phi B\frac{\alpha F_i^*}{F} + \left(\beta_f(\tau_f^*)\gamma_{fm}\frac{F_i^*}{F} + \beta_m(\tau_m^*)\gamma_{mm}\frac{M_i^*}{M}\right)M_s - \delta_m(\tau_m^*)M_i^*.
\end{aligned}
$$

If the mutant strain is rare, the fraction of infecteds are approximately given by $f_i^* = F_i^*/F$ and $m_i^* = M_i^*/M$. If the mutant is rare eqns (3-5) will give the dynamics of the overall system to a good approximation. If the fractions of susceptibles and birth rates settle at equilibrium, the dynamics of the infected resident strain settle at equilibrium and f_i^* and m_i^* are given by:

$$
\left[\begin{array}{c} \dot{f}_i^* \\ \dot{m}_i^* \end{array}\right] = \underbrace{\left[\begin{array}{cc} \beta_f(\tau_f^*)\gamma_{ff}\hat{f}_s - \delta_f(\tau_f^*) + \alpha\hat{B}_f & \beta_m(\tau_m^*)\gamma_{mf}\hat{f}_s \\ \beta_f(\tau_f^*)\gamma_{fmm}\hat{m}_s + \alpha\hat{B}_m & \beta_m(\tau_m^*)\gamma_{mm}\hat{m}_s - \delta_m(\tau_m^*) \end{array}\right]}_{\mathbf{A}^*} \cdot \left[\begin{array}{c} f_i^* \\ m_i^* \end{array}\right]. \qquad (6)
$$

We will calculate the fitness as the number of new infections that an infection with the mutant strain will cause in the next generation of infections. To do so we will use a next generation matrix approach [6]. The next generation matrix facilitates the interpretation of the results; it gives mathematical equivalent results as a direct analysis would [4]. We have summarised the use of the next generation matrix for the calculation of evolutionary singular points, and the analysis of their evolutionary stability in the Appendix of this document.

The Evolutionary Stable Strategy

Next we will determine the evolutionary stable strategies, by means of an evolutionary invasion analysis (see e.g. [5]). To find evolutionary singular strategies, and assess their evolutionary stability it suffices to know the derivatives of the next generation matrix and the left and right eigenvectors of the matrix $\mathbf{A} = \mathbf{A}^*|_{\tau_f^*=\tau_f,\tau_m^*=\tau_m}$. The matrix $\mathbf{A}$ gives the change in the densities at the population dynamical equilibrium. The equilibrium values of the fraction of infected males and females, even when we cannot solve for them in closed form, satisfy

$$
\mathbf{A} \cdot \left(\begin{array}{c} \hat{f}_i \\ \hat{m}_i \end{array}\right) = \left(\begin{array}{c} 0 \\ 0 \end{array}\right).
$$

Therefore the right eigenvector of $\mathbf{A}$ associated with eigenvalue 0 is $\mathbf{w} = \left(\begin{array}{c} \hat{f}_i \\ \hat{m}_i \end{array}\right)$. It is easy to check that the left eigenvector associated with eigenvalue 0 is $\mathbf{v}^T = \left(\frac{\hat{T}_f}{\hat{f}_i}, \frac{\hat{T}_m}{\hat{m}_i}\right)$, where

$$
\hat{T}_f = \frac{\frac{1}{\delta_f - \alpha\hat{B}_f - \beta_f\gamma_{ff}\hat{f}_s}}{\frac{1}{\delta_m - \beta_m\gamma_{mm}\hat{m}_s} + \frac{1}{\delta_f - \alpha\hat{B}_f - \beta_f\gamma_{ff}\hat{f}_s}}.
$$

$$
\hat{T}_m = \frac{\frac{1}{\delta_m - \beta_m\gamma_{mm}\hat{m}_s}}{\frac{1}{\delta_m - \beta_m\gamma_{mm}\hat{m}_s} + \frac{1}{\delta_f - \alpha\hat{B}_f - \beta_f\gamma_{ff}\hat{f}_s}}
$$

A cohort of pathogens infecting females will disappear from the female hosts at rate $\delta_f - \alpha\hat{B}_f - \beta_f\gamma_{ff}\hat{f}_s$ and therefore the pathogen spends on average $(\delta_f - \alpha\hat{B}_f - \beta_f\gamma_{ff}\hat{f}_s)^{-1}$ time in the females, before infecting a male. Similarly, a cohort of pathogens infecting males will spend $(\delta_m - \beta_m\gamma_{mm}\hat{m}_s)^{-1}$ time in the males before infecting a female. The new variables $\hat{T}_f$ and $\hat{T}_m$ therefore carry the interpretation of the fraction of time the infection spends in the male, respectively, female host when the system is at equilibrium. Note that for this choice of eigenvectors $\left(\frac{\hat{T}_f}{\hat{f}_i}, \frac{\hat{T}_m}{\hat{m}_i}\right) \cdot (\hat{f}_i, \hat{m}_i)^T = 1$.

For the next generation approach we need to specify the matrices $\mathbf{K}^*$ and $\mathbf{V}^*$. Many choices are possible, here we found following choice of matrices convenient:

$$\mathbf{K}^* = \begin{bmatrix} \beta_f(\tau_f^*)\gamma_{ff}\hat{f}_s & \beta_m(\tau_m^*)\gamma_{mf}\hat{f}_s \\ \beta_f(\tau_f^*)\gamma_{fm}\hat{m}_s & \beta_m(\tau_m^*)\gamma_{mm}\hat{m}_s \end{bmatrix}$$

and

$$\mathbf{V}^* = \begin{bmatrix} \delta_f(\tau_f^*) - \alpha\hat{B}_f & 0 \\ -\alpha\hat{B}_m & \delta_m(\tau_m^*) \end{bmatrix}.$$

These choices allow us to calculate the fitness as the dominant eigenvalue of $\mathbf{K}^*\mathbf{V}^{*-1}$. From the fitness we can calculate the selection gradient, which is given by

$$\left.\frac{\partial\lambda}{\partial\xi^*}\right|_{\xi^*=\xi} = \frac{\mathbf{v}^T \left.\left(\frac{\partial\mathbf{K}^*}{\partial\xi^*} - \frac{\partial\mathbf{V}^*}{\partial\xi^*}\right)\right|_{\xi^*=\xi}\mathbf{w}}{\mathbf{v}^T\mathbf{V}\mathbf{w}},$$

where $\mathbf{V} = \mathbf{V}^*|_{\tau_f^*=\tau_f,\tau_m^*=\tau_m}$. The selection gradient is useful because at the evolutionary stable strategies the selection gradient is zero. We will calculate the selection gradient for two different scenarios: the cases where exploitation strategy is host specific and there are two traits $\xi = \tau_f$ and $\xi = \tau_m$, and the case where the pathogen cannot separately control the exploitation in the sexes separately and there is only one trait $\xi = \tau_f = \tau_m$.

Evolution when the host exploitation strategy is not sex-specific

When the exploitation strategy is not sex-specific we choose $\tau = \tau_f = \tau_m$ and $\tau^* = \tau_f^* = \tau_m^*$. We now find that

$$\left.\frac{\partial\mathbf{K}^*}{\partial\tau^*}\right|_{\tau^*=\tau} = \mathbf{K}\begin{bmatrix} \frac{\beta_f'}{\beta_f} & 0 \\ 0 & \frac{\beta_m'}{\beta_m} \end{bmatrix}$$

where $\beta_a' = \left.\frac{\beta_a(\tau_a^*)}{\partial\tau_a^*}\right|_{\tau_a{}^*=\tau_a}$ with $a \in \{f, m\}$, and that therefore

$$\frac{\mathbf{v}^T \left.\left(\frac{\partial\mathbf{K}^*}{\partial\tau^*}\right)\right|_{\tau^*=\tau}\mathbf{w}}{\mathbf{v}^T\mathbf{V}\mathbf{w}} = \frac{\mathbf{v}^T\mathbf{V}\begin{bmatrix} \frac{\beta_f'}{\beta_f} & 0 \\ 0 & \frac{\beta_m'}{\beta_m} \end{bmatrix}\mathbf{w}}{\mathbf{v}^T\mathbf{V}\mathbf{w}} = c_m\frac{\beta_m'}{\beta_m} + c_f\frac{\beta_f'}{\beta_f}$$

where $c_m = \frac{\hat{T}_m\delta_m(\tau_m)}{\mathbf{v}^T\mathbf{V}\mathbf{w}}$ and $c_f = \frac{\hat{T}_f\delta_f(\tau_f) - \alpha(\hat{T}_f\hat{B}_f + T_m\hat{B}_m\hat{f}_i/\hat{m}_i)}{\mathbf{v}^T\mathbf{V}\mathbf{w}}$ and note that $c_m + c_f = 1$. The derivative of $\mathbf{V}^*$ is:

$$\left.\frac{\partial\mathbf{V}^*}{\partial\tau^*}\right|_{\tau^*=\tau} = \begin{bmatrix} \delta_f' & 0 \\ 0 & \delta_m' \end{bmatrix}.$$

The selection gradient then is:

$$\left.\frac{\partial\lambda}{\partial\tau^*}\right|_{\tau^*=\tau} = c_m\frac{\beta_m'}{\beta_m} + c_f\frac{\beta_f'}{\beta_f} - \frac{\hat{T}_f\delta_f' + \hat{T}_m\delta_m'}{\hat{T}_f\delta_f + \hat{T}_m\delta_m - \alpha(\hat{T}_f\hat{B}_f + \hat{T}_m\hat{B}_m\frac{\hat{f}_i}{\hat{m}_i})}.$$

The evolutionarily singular strategy $\tilde{\tau}$ can then be identified from $\left.\frac{\partial\lambda}{\partial\tau^*}\right|_{\tau^*=\tau=\tilde{\tau}} = 0$. This is an evolutionarily stable strategy if:

$$\frac{\mathbf{v}^T \left.\frac{\partial^2\mathbf{K}^*\mathbf{V}^{*-1}}{\partial\tau^{*2}}\right|_{\tau^*=\tau=\tilde{\tau}}\mathbf{V}\mathbf{w}}{\mathbf{v}^T\mathbf{V}\mathbf{w}} = c_m\frac{\beta_m''}{\beta_m} + c_f\frac{\beta_f''}{\beta_f} - \frac{\hat{T}_f\delta_f'' + \hat{T}_m\delta_m''}{\hat{T}_f\delta_f + \hat{T}_m\delta_m - \alpha(\hat{T}_f\hat{B}_f + \hat{T}_m\hat{B}_m\frac{\hat{f}_i}{\hat{m}_i})} < 0.$$

With these expressions the evolutionarily singular strategies can be calculated and their evolutionary stability can be assessed.

To interpret these expression, the following definitions are useful. Let the average transmission rate be defined as

$$\bar{\beta}(\tau^*) = \beta_f(\tau^*)^{c_f}\beta_m(\tau^*)^{c_m}.$$

Note that the dependence on τ^* is only through the transmission rates β_f and β_m, the constants c_f and c_m are independent of τ^*. Let the average removal rate be given by

$$\bar{\delta}(\tau^*) = \mathbf{v}^T\mathbf{V}^*\mathbf{w} = \hat{T}_f\delta_f(\tau^*) + \hat{T}_m\delta_m(\tau^*) - \alpha(\hat{T}_f\hat{B}_f + \hat{T}_m\hat{B}_m\frac{\hat{f}_i}{\hat{m}_i})$$

where also here the dependence on τ^* is only through the recovery rates δ_f and δ_m, but the birth rates $\hat{B}_f$ and $\hat{B}_m$ and the quantities $\hat{T}_f$ and $\hat{T}_m$ are evaluated at $\tilde{\tau}$ and independent of τ^*. Using these averages we can rewrite the condition for the evolutionarily singular strategy as:

$$0 = \left.\frac{\partial\bar{\beta}(\tau^*)}{\bar{\beta}(\tau^*)\partial\tau^*}\right|_{\tau^*=\tilde{\tau}} - \left.\frac{\partial\bar{\delta}(\tau^*)}{\bar{\delta}(\tau^*)\partial\tau^*}\right|_{\tau^*=\tilde{\tau}}.$$

By defining the (local) inverse g of $\bar{\delta}$, such that $g(\bar{\delta}(\tau^*)) = \tau^*$, we can write $\bar{\beta}$ as a function of $\bar{\delta}$: $\bar{\beta}(\tau^*) = \bar{\beta}(g(\bar{\delta}))$. we can thus rewrite the condition for the evolutionarily singular strategy as:

$$0 = \left.\frac{\partial\bar{\delta}(\tau^*)}{\bar{\beta}(g(\bar{\delta}))\partial\tau^*}\right|_{\tau^*=\tilde{\tau}} \left(\frac{\mathrm{d}\bar{\beta}(g(\bar{\delta}))}{\mathrm{d}\bar{\delta}} - \frac{\bar{\beta}(g(\bar{\delta}))}{\bar{\delta}}\right)$$

The functions $\bar{\beta}(\tau^*)$ and $\bar{\delta}(\tau^*)$ together define a parametric curve in the two dimensional space spanned by $\bar{\delta}$ and $\bar{\beta}$. The above condition shows that at the evolutionarily singular strategy, where $\tau^* = \tilde{\tau}$ this curve has as tangent with gradient $\frac{\bar{\beta}}{\bar{\delta}}$. At the evolutionarily singular point the tangent to the curve will go through the origin. This generalises a result by van Baalen and Sabelis [7] where the same observation was made for a the evolution of virulence in a simple SIR model.

The second derivative of the parametric curve with respect to $\bar{\delta}$ is given by:

$$\frac{\mathrm{d}^2\bar{\beta}(g(\bar{\delta}))}{\mathrm{d}\bar{\delta}^2} = \left(\frac{\partial\bar{\delta}(\tau^*)}{\partial\tau^*}\right)^{-2}\left(\frac{\partial^2\bar{\beta}(\tau^*)}{\partial\tau^{*2}} - \frac{\frac{\partial\bar{\beta}(\tau^*)}{\partial\tau^*}\frac{\partial^2\bar{\delta}(\tau^{*2})}{\partial\tau^*}}{\frac{\partial\bar{\delta}(\tau^*)}{\partial\tau^*}}\right).$$

At the evolutionarily singular point it is:

$$\begin{aligned}\frac{\mathrm{d}^2\bar{\beta}(g(\bar{\delta}))}{\mathrm{d}\bar{\delta}^2} &= \bar{\beta}(\tilde{\tau})\left(\frac{\partial\bar{\delta}(\tau^*)}{\partial\tau^*}\right)^{-2}\left.\left(\frac{\partial^2\bar{\beta}(\tau^*)}{\bar{\beta}(\tilde{\tau})\partial\tau^{*2}} - \frac{\partial^2\bar{\delta}(\tau^{*2})}{\bar{\delta}(\tilde{\tau})\partial\tau^*}\right)\right|_{\tau^*=\tilde{\tau}} \\ &\propto c_m\frac{\beta''_m}{\beta_m} + c_f\frac{\beta''_f}{\beta_f} - c_mc_f\left(\frac{\beta'_m}{\beta_m} - \frac{\beta'_f}{\beta_f}\right)^2 - \frac{\hat{T}_f\delta''_f + \hat{T}_m\delta''_m}{\hat{T}_f\delta_f + \hat{T}_m\delta_m - \alpha(\hat{T}_f\hat{B}_f + \hat{T}_m\hat{B}_m\frac{\hat{f}_i}{\hat{m}_i})}\end{aligned}$$

It follows that, provided we have chosen our traits such that $\frac{\partial\bar{\delta}(\tau^*)}{\partial\tau^*} > 0$, that the singular point cannot be evolutionary stable if the parametric curve is convex at the singular point, and for evolutionary stability concavity is a necessary requirement. If $\frac{\beta'_m}{\beta_m} \neq \frac{\beta'_f}{\beta_f}$ it is possible though that the non-specific singular point is evolutionary unstable even if the parametric curve is concave.

Evolution when the host exploitation strategy is sex-specific

When the exploitation strategy is sex-specific we have two traits that can evolve, one for the traits expressed in males, one in females. We will therefore consider a vector of traits $\boldsymbol{\tau} = (\tau_m, \tau_f)^T$ and $\boldsymbol{\tau}^* = (\tau_m{}^*, \tau_f{}^*)^T$. We now find that the selection gradient is:

$$\left.\frac{\partial\lambda}{\partial\boldsymbol{\tau}^*}\right|_{\boldsymbol{\tau}^*=\boldsymbol{\tau}} = \begin{pmatrix} c_m\frac{\beta'_m}{\beta_m} \\ c_f\frac{\beta'_f}{\beta_f}\end{pmatrix} - \frac{1}{\mathbf{v}^T\mathbf{V}\mathbf{w}}\begin{pmatrix}\hat{T}_m\delta'_m \\ \hat{T}_f\delta'_f\end{pmatrix}.$$

The evolutionarily singular strategy $\tilde{\boldsymbol{\tau}}$ can then be identified from

$$\left.\frac{\partial\lambda}{\partial\boldsymbol{\tau}^*}\right|_{\boldsymbol{\tau}^*=\boldsymbol{\tau}=\tilde{\boldsymbol{\tau}}} = \begin{pmatrix}0\\0\end{pmatrix}.$$

This is an evolutionarily stable strategy if:

$$c_f\frac{\beta''_f}{\beta_f} - \frac{\hat{T}_f\delta''_f}{\hat{T}_f\delta_f + \hat{T}_m\delta_m - \alpha(\hat{T}_f\hat{B}_f\frac{\hat{m}_i}{\hat{f}_i} + \hat{T}_m\hat{B}_m)} < 0$$

and

$$c_m \frac{\beta''_m}{\beta_m} - \frac{\hat{T}_m \delta''_m}{\hat{T}_f \delta_f + \hat{T}_m \delta_m - \alpha(\hat{T}_f \hat{B}_f \frac{\hat{m}_i}{\hat{f}_i} + \hat{T}_m \hat{B}_m)} < 0.$$

(The left hand sides of the inequalities are the eigenvalues of the matrix with the second order derivatives, see [8]).

No physiological difference between the sexes

The above calculations allow the identification of the evolutionarily stable virulence rates in general. A special case is when there are no physiological differences between the sexes. This is of interest when we would like to know if sex-specific virulence can evolve. The case when there are no physiological differences between the sexes is the most difficult scenario for sex-specific exploitation strategies to evolve: if there are differences between the sexes can evolve when the sexes are physiologically equal, they are also likely to evolve if there physiological differences between the sexes.

For this scenario we choose $\beta_f(\tau) = \beta_m(\tau) = \beta(\tau)$ and $\delta_f(\tau) = \delta_m(\tau) = \delta(\tau)$, which can be achieved by choosing $\sigma_f(\tau) = \sigma_m(\tau) = \sigma(\tau)$, $\nu_f(\tau) = \nu_m(\tau) = \nu(\tau)$ and $\mu_f = \mu_m = \mu$. We will identify the evolutionarily stable states by evaluating this fitness measure in two special cases of interest, namely when the virulence is not sex-specific and a single trait controls the virulence in females and males so that $\tau_f = \tau_m = \tau$, and after that we consider the case where virulence is sex-specific and the virulence in females and males can be controlled separately by the parameters τ_f and τ_m.

No physiological differences and host exploitation is not sex-specific. When the exploitation strategy is not sex-specific we choose $\tau = \tau_f = \tau_m$ and $\tau^* = \tau^*_f = \tau^*_m$. Using our results above we find that the selection gradient then is:

$$\left.\frac{\partial \lambda}{\partial \tau^*}\right|_{\tau^*=\tau} = \frac{\beta'}{\beta} - \frac{\delta'}{\delta - \alpha(\hat{T}_f \hat{B}_f + \hat{T}_m \hat{B}_m \frac{\hat{f}_i}{\hat{m}_i})}.$$

and he evolutionarily singular strategy $\tilde{\tau}$ is

$$\frac{\beta'}{\beta} - \frac{\delta'}{\delta - \alpha \hat{T}_f(\hat{B}_f + \hat{R}_{mf} \hat{B}_m)} = 0.$$

where

$$\hat{R}_{mf} = \frac{\hat{T}_m \hat{f}_i}{\hat{T}_f \hat{m}_i} = \frac{\beta \gamma_{mf} \hat{f}_s}{\tilde{\delta} - \beta \gamma_{mm} \hat{m}_s}$$

The parametric curve that is obtained by plotting $\beta(\tau)$ against $\delta(\tau)$ has a tangent at the point $(\delta(\tilde{\tau}), \beta(\tilde{\tau}))$ that goes through the point $(\alpha \hat{T}_f(\hat{B}_f + \hat{R}_{mf} \hat{B}_m), 0)$ [9]. This is the basis for the graphical construction used in the main text.

The parameter $\hat{R}_{mf}$ is the expected size of the next generation (in terms of pathogen infection) of infected females that originate from one infected son. This measure including all the females that originate other males infected through a male line by this son. If the chance of a male infecting a male is $\frac{\beta(\tilde{\delta})\gamma_{mm}\hat{m}_s}{\tilde{\delta}}$ the total number of infected females is:

$$\hat{R}_{mf} = \frac{\beta(\tilde{\delta})\gamma_{mf}\hat{f}_s}{\tilde{\delta}} \sum_{i=0}^{\infty} \left(\frac{\beta(\tilde{\delta})\gamma_{mm}\hat{m}_s}{\tilde{\delta}} \right)^i = \frac{\beta(\tilde{\delta})\gamma_{mf}\hat{f}_s}{\tilde{\delta} - \beta(\tilde{\delta})\gamma_{mm}\hat{m}_s}.$$

This is the "reproductive value" of a pathogen in a fertile female host, where the currency of the value is the number of female hosts that can pass the infection on.

This is an evolutionarily stable strategy if:

$$\frac{\beta''}{\beta} - \frac{\delta''}{\delta - \hat{T}_f \alpha(\hat{B}_f + \hat{R}_{mf} \hat{B}_m)} < 0.$$

The functions $\beta(\tau)$ and $\delta(\tau)$ define a parametric curve in the two dimensional space spanned by $\beta(\tau)$ and $\delta(\tau)$. The singular point is evolutionary stable if the parametric curve is concave at the singular point.

Next we consider the case where virulence is sex-specific and controlled by two traits τ_f and τ_m. To find the evolutionarily stable host exploitation strategy we consider the traits that control the host exploitation in males and females separately.

No physiological differences and host exploitation is male specific. We can find the selection gradient of a mutant with a marginally different value of τ_m as:

$$\left.\frac{\partial\lambda}{\partial{\tau_m}^*}\right|_{{\tau_m}^*=\tau_m} = \frac{\hat{T}_m\delta(\tau_m)}{\mathbf{v}^T\mathbf{V}\mathbf{w}}\frac{\beta'(\tau_m)}{\beta(\tau_m)} - \frac{\hat{T}_m\delta'(\tau_m)}{\mathbf{v}^T\mathbf{V}\mathbf{w}}.$$

As the selection gradient does not only depend on τ_m we can now solve for the value of $\tilde{\tau}_m$ that makes the selection gradient zero we get the singular strategy:

$$\beta'(\tilde{\tau}_m) = \frac{\beta(\tilde{\tau}_m)}{\tilde{\tau}_m}. \tag{7}$$

Such singular strategy is evolutionarily stable when:

$$\frac{\beta''(\tilde{\tau}_m)}{\beta(\tau_m)} < \frac{\delta''(\tilde{\tau}_m)}{\delta(\tau_m)}. \tag{8}$$

This stability condition requires that the trade-off function is concave.

No physiological differences and host exploitation is female specific. We can find the selection gradient of a mutant with a marginally different value of τ_f as:

$$\left.\frac{\partial\lambda}{\partial{\tau_f}^*}\right|_{{\tau_f}^*=\tau_f} = \frac{\hat{T}_f\delta(\tau_f) - \alpha(\hat{T}_f\hat{B}_f + T_m\hat{B}_m\hat{f}_i/\hat{m}_i)}{\mathbf{v}^T\mathbf{V}\mathbf{w}}\frac{\beta'(\tau_f)}{\beta(\tau_f)} - \frac{\hat{T}_f\delta'(\tau_f)}{\mathbf{v}^T\mathbf{V}\mathbf{w}}.$$

Assuming that the male trait is set at $\tilde{\tau}_m$ we can now solve for the value of $\tilde{\tau}_f$ that makes the selection gradient zero we get the singular strategy:

$$\beta'(\tilde{\tau}_f) = \frac{\beta(\tilde{\tau}_f)}{\tilde{\delta}_f - \alpha\left(\hat{B}_f + \hat{R}_{mf}\hat{B}_m\right)}. \tag{9}$$

Such singular strategy is evolutionarily stable when:

$$\frac{\beta''}{\beta} - \frac{\delta''}{\delta - \alpha(\hat{B}_f + \hat{R}_{mf}\hat{B}_m)} < 0. \tag{10}$$

This stability condition requires that the trade-off function is concave at $\tilde{\tau}_m$.

Numerically solving the ESS values

Although our method shows how to derive the fitness, selection gradient and ESS values, and we can glean some generally properties from these results, in order to obtain the values of the ESS exploitation strategies, in most cases this will have to be done numerically. Generally the obstacle in these calculations is to find the equilibrium values of the epidemiological variables, which for this class of models can mostly not be found in closed form.

We use a numerical routine to find the ESS values. To do this the following steps have to be taken:

-Setting parameters and define trade offs

-Numerically establishing the equilibrium values. This is a function of the value of the trait.

-Checking if the equilibrium is non-trivial.

-Calculating the selection gradient. This is a function of the trait, and the equilibrium values.

-Numerically locate the trait value for which the selection gradient vanishes.

This routine was implemented in Mathematica using a regula falsi routine to find the zero of the selection gradient. Numerical exploration revealed that for trade off which lead to low ESS virulence levels, in which the probability of dying for the male strategy did not exceed 25%

the results depended mainly on the amount of vertical transmission: the ESS levels were fairly insensitive to variation in the epidemiological parameters. Results representative of this scenario are depicted in Fig. 4 of the main paper. This scenario applies to the pathogens discussed in the paper, where the probability of dying from the disease is appreciable, but not not exceeding 25%.

If the trade offs are chosen such that they lead to very high virulence levels (probability of dying of the disease exceeding 50%) the results are more sensitive to other parameters, but we also found the difference in the probability of dying of the disease becomes very similar for males and females for sex-specific strategies, particular if the probability of vertical transmission is smallish. This effect will be even more pronounced in if sexual maturity is taken into account. For diseases that can kill before sexual maturity, children infected by their mother will have a very small chance to contribute to the vertical transmission of the disease. In such cases the effect of vertical transmission on the evolution of the sex-specific virulence of the disease is expected to be small and the male and female specific strategies to be very similar.

The Next Generation approach to calculating fitness

The basis of the next generation approach is to define two new matrices, $\mathbf{K}^*$ and $\mathbf{V}^*$ such that $\mathbf{A}^* = \mathbf{K}^* - \mathbf{V}^*$. The next generation approach exploits the equivalence that when matrix $\mathbf{A}^*$ has a positive eigenvalue, the matrix $\mathbf{K}^*{\mathbf{V}^*}^{-1}$ has an eigenvalue that exceeds one [6]. Therefore the dominant eigenvalue of the next generation matrix $\mathbf{K}^*{\mathbf{V}^*}^{-1}$ is an appropriate fitness measure. For this to work $\mathbf{V}^*$ needs to be invertible, and to allow for the interpretation in terms of generations the real parts of all its eigenvalues should be positive [4].

The dominant eigenvalue of $\mathbf{A}^*$ describes the rate of growth of a rare mutant population over time. In the next generation approach the rare mutant infections are counted per generation and the dominant eigenvalue of $\mathbf{K}^*{\mathbf{V}^*}^{-1}$ is the multiplication factor by which each generation differs from the previous generation, and therefore the average number of offspring. As fitness as often defined in terms of the number of offspring, this is a convenient fitness measure that aligns with the biological interpretation. Note though, that there are many different ways in which we can define a generation (which in our model amount to different choices of the matrices $\mathbf{K}^*$ and $\mathbf{V}^*$), and therefore what constitutes offspring. For the particular choice of $\mathbf{K}^*$ and $\mathbf{V}^*$ made here a generation runs from one horizontal transmission event to the next. If the pathogen transmits vertically between two horizontal transmission events the vertical infections are considered to be part of the same generation as the mother's infection.

To calculate the fitness, one can reason as follows: let ${\mathbf{v}^*}^T$ and $\mathbf{u}^*$ be left and right eigenvectors of the next generation matrix $\mathbf{K}^*{\mathbf{V}^*}^{-1}$ associated with the dominant eigenvalue. We can find the eigenvalue from [4,10] :

$$\lambda = \frac{{\mathbf{v}^*}^T \mathbf{K}^* {\mathbf{V}^*}^{-1} \mathbf{u}^*}{{\mathbf{v}^*}^T \mathbf{u}^*},$$

which allows calculating the fitness once the left and right eigenvectors are known.

We will now identify the evolutionarily stable points. To do so we will assume that the mutant we study has trait ξ^*, whilst the resident has trait ξ.[2] If the traits of the mutant pathogen are equal to that of the resident we have $\xi^* = \xi$. We denote $\mathbf{A} = \mathbf{A}^*|_{\xi^*=\xi.}$. The matrix $\mathbf{A}$ gives the invasion of a mutant with trait $\xi^* = \xi$. The matrix $\mathbf{A}$ can also be retrieved from the differential equations for f_i and m_i specified in (3) and (4), by substituting the equilibrium values $\hat{B}_m$, $\hat{B}_f$, $\hat{f}_s$ and $\hat{m}_s$. The matrix $\mathbf{A}$ has, therefore, a right eigenvector $\mathbf{w}$ which contains the value of the resident variables $\hat{f}_i$ and $\hat{m}_i$ at equilibrium and which satisfies $\mathbf{A}\mathbf{w} = (0,0)^T$, and hence the matrix $\mathbf{A}$ has one eigenvalue equal to zero. Let the left eigenvector of $\mathbf{A}$ that is associated with eigenvalue zero be $\mathbf{v}^T$ such that $\mathbf{v}^T\mathbf{A} = (0,0)$. If we denote $\mathbf{K} = \mathbf{K}^*|_{\xi^*=\xi}$ and $\mathbf{V} = \mathbf{V}^*|_{\xi^*=\xi}$ it is easy to show that $\mathbf{v}^T$ is also a left eigenvector of the next generation matrix $\mathbf{K}\mathbf{V}^{-1}$: this follows because $\mathbf{v}^T\mathbf{A} = \mathbf{v}^T(\mathbf{K} - \mathbf{V}) = 0$ and hence $\mathbf{v}^T\mathbf{K}\mathbf{V}^{-1} = \mathbf{v}$. Thus if $\mathbf{A}$ has an eigenvalue zero, then $\mathbf{K}\mathbf{V}^{-1}$ has eigenvalue 1. The right eigenvector of $\mathbf{K}\mathbf{V}^{-1}$ associated with the eigenvalue 1 we denote $\mathbf{u}$ and we can easily compute the eigenvector $\mathbf{u}$ as $\mathbf{u} = \mathbf{V}\mathbf{w}$. The vector $\mathbf{u}$ is an eigenvalue of $\mathbf{K}\mathbf{V}^{-1}$:

[2] in the context of the sex specific virulence, we can consider the traits that control virulence to be functions of ξ and ξ^*, and can write these traits as $\tau_f(\xi)$, $\tau_m(\xi)$ and $\tau_f^*(\xi^*)$, $\tau_m^*(\xi^*)$.

because $\mathbf{K}\mathbf{V}^{-1}\mathbf{u} = \mathbf{K}\mathbf{V}^{-1}\mathbf{V}\mathbf{w} = \mathbf{K}\mathbf{w}$, and because $\mathbf{A}\mathbf{w} = (\mathbf{K} - \mathbf{V})\mathbf{w} = 0$, $\mathbf{K}\mathbf{w} = \mathbf{V}\mathbf{w} = \mathbf{u}$. Hence $\mathbf{K}\mathbf{V}^{-1}\mathbf{u} = \mathbf{u}$.

It follows from the argument above that the fitness is unity when $\xi^* = \xi$ (and this makes perfect biological sense as it tells us that when the mutant and resident have the same trait, the fitness is one and the mutant is neutral with respect to invasion).

We can now calculate the selection gradient as:

$$\left.\frac{\partial\lambda}{\partial\xi^*}\right|_{\xi^*=\xi} = \frac{\mathbf{v}^T \left.\frac{\partial\mathbf{K}^*\mathbf{V}^{*-1}}{\partial\xi^*}\mathbf{u}\right|_{\xi^*=\xi}}{\mathbf{v}^T\mathbf{u}} = \frac{\mathbf{v}^T \left.\left(\frac{\partial\mathbf{K}^*}{\partial\xi^*} - \frac{\partial\mathbf{V}^*}{\partial\xi^*}\right)\mathbf{w}\right|_{\xi^*=\xi}}{\mathbf{v}^T\mathbf{V}\mathbf{w}}.$$

(see [4]).[3] Any ESS value $\tilde{\xi}$ must therefore satisfy

$$\left.\frac{\partial\lambda}{\partial\xi^*}\right|_{\xi^*=\xi=\tilde{\xi}} = \frac{\mathbf{v}^T \left.\frac{\partial\mathbf{K}^*\mathbf{V}^{*-1}}{\partial\xi^*}\right|_{\xi^*=\xi=\tilde{\xi}}\mathbf{u}}{\mathbf{v}^T\mathbf{u}} = 0 \tag{11}$$

From this we can can glean that the matrix $\left.\frac{\partial\mathbf{K}^*\mathbf{V}^{*-1}}{\partial\xi^*}\right|_{\xi^*=\xi=\tilde{\xi}}$ has an eigenvalue zero with left and right eigenvalues $\mathbf{v}^T$ and $\mathbf{u}$.

For an ESS it is furthermore required that

$$\left.\frac{\partial^2\lambda}{\partial{\xi^*}^2}\right|_{\xi^*=\xi=\tilde{\xi}} < 0.$$

Working out the second derivative gives

$$\begin{aligned}\frac{\partial^2\lambda}{\partial{\xi^*}^2} = {} & \frac{\frac{\partial^2\mathbf{v}^{*T}}{\partial^2\xi^*}\mathbf{K}^*\mathbf{V}^{*-1}\mathbf{u}^*}{\mathbf{v}^{*T}\mathbf{u}^*} + 2\frac{\frac{\partial\mathbf{v}^{*T}}{\partial\xi^*}\frac{\partial\mathbf{K}^*\mathbf{V}^{*-1}}{\partial\xi^*}\mathbf{u}^*}{\mathbf{v}^{*T}\mathbf{u}^*} + \frac{\mathbf{v}^{*T}\frac{\partial^2\mathbf{K}^*\mathbf{V}^{*-1}}{\partial{\xi^*}^2}\mathbf{u}^*}{\mathbf{v}^{*T}\mathbf{u}^*} + 2\frac{\mathbf{v}^{*T}\frac{\partial\mathbf{K}^*\mathbf{V}^{*-1}}{\partial\xi^*}\frac{\partial\mathbf{u}^*}{\partial\xi^*}}{\mathbf{v}^{*T}\mathbf{u}^*} \\ & + \frac{\mathbf{v}^{*T}\mathbf{K}^*\mathbf{V}^{*-1}\frac{\partial^2\mathbf{u}^*}{\partial{\xi^*}^2}}{\mathbf{v}^{*T}\mathbf{u}^*} + 2\frac{\frac{\partial\mathbf{v}^{*T}}{\partial\xi^*}\mathbf{K}^*\mathbf{V}^{*-1}\frac{\partial\mathbf{u}^*}{\partial\xi^*}}{\mathbf{v}^{*T}\mathbf{u}^*} - \frac{\partial\lambda}{\partial\xi^*}\frac{\frac{\partial\mathbf{v}^{*T}}{\partial\xi^*}\mathbf{u}^* + \mathbf{v}^{*T}\frac{\partial\mathbf{u}^*}{\partial\xi^*}}{\mathbf{v}^{*T}\mathbf{u}^*} \\ & - \lambda\frac{\frac{\partial^2\mathbf{v}^{*T}}{\partial^2\xi^*}\mathbf{u}^* + 2\frac{\partial\mathbf{v}^{*T}}{\partial\xi^*}\frac{\partial\mathbf{u}^*}{\partial\xi^*} + \mathbf{v}^{*T}\frac{\partial^2\mathbf{u}^*}{\partial\xi^*}^2}{\mathbf{v}^{*T}\mathbf{u}^*}.\end{aligned}$$

Using the fact that $\frac{\partial\mathbf{K}^*\mathbf{V}^{*-1}\mathbf{u}^*}{\partial\xi^*} = \frac{\partial\lambda}{\partial\xi^*} + \lambda\frac{\partial\mathbf{u}^*}{\partial\xi^*}$ this simplifies to

$$\begin{aligned}\frac{\partial^2\lambda}{\partial{\xi^*}^2} = {} & \frac{\frac{\partial^2\mathbf{v}^{*T}}{\partial^2\xi^*}\mathbf{K}^*\mathbf{V}^{*-1}\mathbf{u}^*}{\mathbf{v}^{*T}\mathbf{u}^*} + \frac{\mathbf{v}^{*T}\frac{\partial^2\mathbf{K}^*\mathbf{V}^{*-1}}{\partial{\xi^*}^2}\mathbf{u}^*}{\mathbf{v}^{*T}\mathbf{u}^*} + 2\frac{\mathbf{v}^{*T}\frac{\partial\mathbf{K}^*\mathbf{V}^{*-1}}{\partial\xi^*}\frac{\partial\mathbf{u}^*}{\partial\xi^*}}{\mathbf{v}^{*T}\mathbf{u}^*} \\ & + \frac{\mathbf{v}^{*T}\mathbf{K}^*\mathbf{V}^{*-1}\frac{\partial^2\mathbf{u}^*}{\partial{\xi^*}^2}}{\mathbf{v}^{*T}\mathbf{u}^*} + \frac{\partial\lambda}{\partial\xi^*}\frac{\frac{\partial\mathbf{v}^{*T}}{\partial\xi^*}\mathbf{u}^* - \mathbf{v}^{*T}\frac{\partial\mathbf{u}^*}{\partial\xi^*}}{\mathbf{v}^{*T}\mathbf{u}^*} - \lambda\frac{\frac{\partial^2\mathbf{v}^{*T}}{\partial^2\xi^*}\mathbf{u}^* + \mathbf{v}^{*T}\frac{\partial^2\mathbf{u}^*}{\partial\xi^*}^2}{\mathbf{v}^{*T}\mathbf{u}^*}.\end{aligned}$$

When $\xi = \xi^*$ this simplifies to:

$$\left.\frac{\partial^2\lambda}{\partial{\xi^*}^2}\right|_{\xi^*=\xi} = \left.\frac{\mathbf{v}^T\frac{\partial^2\mathbf{K}^*\mathbf{V}^{*-1}}{\partial{\xi^*}^2}\mathbf{u}}{\mathbf{v}^T\mathbf{u}}\right|_{\xi^*=\xi} + 2\left.\frac{\mathbf{v}^T\frac{\partial\mathbf{K}^*\mathbf{V}^{*-1}}{\partial\xi^*}\frac{\partial\mathbf{u}^*}{\partial\xi^*}}{\mathbf{v}^T\mathbf{u}}\right|_{\xi^*=\xi} + \left.\frac{\partial\lambda}{\partial\xi^*}\frac{\frac{\partial\mathbf{v}^{*T}}{\partial\xi^*}\mathbf{u} - \mathbf{v}^T\frac{\partial\mathbf{u}^*}{\partial\xi^*}}{\mathbf{v}^T\mathbf{u}}\right|_{\xi^*=\xi}.$$

Finally, when $\xi = \xi^* = \tilde{\xi}$, at which point $\left.\frac{\partial\lambda}{\partial\xi^*}\right|_{\xi^*=\xi=\tilde{\xi}} = 0$, and also $\mathbf{v}$ is a left eigenvector to eigenvalue zero of the matrix $\left.\frac{\partial\mathbf{K}^*\mathbf{V}^{*-1}}{\partial\xi^*}\right|_{\xi^*=\xi=\tilde{\xi}}$ we find:

$$\left.\frac{\partial^2\lambda}{\partial{\xi^*}^2}\right|_{\xi^*=\xi=\tilde{\xi}} = \frac{\mathbf{v}^T\left.\frac{\partial^2\mathbf{K}^*\mathbf{V}^{*-1}}{\partial{\xi^*}^2}\right|_{\xi^*=\xi=\tilde{\xi}}\mathbf{u}}{\mathbf{v}^T\mathbf{u}}$$

[3]To see this, realise that $\left.\frac{\partial\mathbf{v}^{*T}}{\partial\xi^*}\right|_{\xi^*=\xi}\mathbf{K}\mathbf{V}^{-1}\mathbf{u} = \left.\frac{\mathbf{v}^{*T}}{\partial\xi^*}\right|_{\xi^*=\xi}\mathbf{u}$ and $\mathbf{v}^T\mathbf{K}\mathbf{V}^{-1}\left.\frac{\partial\mathbf{u}^*}{\partial\xi^*}\right|_{\xi^*=\xi} = \mathbf{v}^T\left.\frac{\partial\mathbf{u}^*}{\partial\xi^*}\right|_{\xi^*=\xi}$

The criterion for evolutionary stability

$$\left.\frac{\partial^2 \lambda}{\partial {\xi^*}^2}\right|_{\xi^*=\xi=\tilde{\xi}} < 0.$$

is thus equivalent to

$$\frac{\mathbf{v}^T \left.\frac{\partial^2 \mathbf{K}^* {\mathbf{V}^*}^{-1}}{\partial {\xi^*}^2}\right|_{\xi^*=\xi=\tilde{\xi}} \mathbf{u}}{\mathbf{v}^T \mathbf{u}} < 0 \tag{12}$$

To find the evolutionary stable strategies, it suffices to know the eigenvectors $\mathbf{v}$ and $\mathbf{u}$ of the next generation matrix for a neutral mutant, and its first two derivatives with respect to the mutant's trait.

Supplementary References

[1] Anderson, R. M. and May, R. M. 1992. Infectious diseases of humans: dynamics and control (Vol. 28). Oxford University Press. Oxford.

[2] Alizon, S., Hurford, A., Mideo, N. and Van Baalen, M., 2009. Virulence evolution and the trade-off hypothesis: history, current state of affairs and the future. *Journal of Evolutionary Biology*, 22(2), pp.245-259.

[3] Metz, J.A.J., Nisbet, R.M. and Geritz, S.A., 1992. How should we define fitness for general ecological scenarios?. *Trends in Ecology & Evolution*, 7(6), pp.198-202

[4] Hurford, A., Cownden, D. and Day, T., 2010. Next-generation tools for evolutionary invasion analyses. *Journal of the Royal Society Interface*, 7, pp.561-571.

[5] Otto, S.P. and Day, T., 2007. A biologist's guide to mathematical modeling in ecology and evolution. Princeton University Press. Princeton.

[6] Diekmann, O., Heesterbeek, J.A.P. and Roberts, M.G., 2009. The construction of next-generation matrices for compartmental epidemic models. *Journal of the Royal Society Interface*, p.rsif20090386.

[7] Van Baalen, M. and Sabelis, M.W., 1995. The dynamics of multiple infection and the evolution of virulence. *The American Naturalist*, pp.881-910.

[8] Leimar, O., 2005. Multidimensional convergence stability and the canonical adaptive dynamics. Elements of Adaptive Dynamics, pp.117-128.
http://www.zoologi.su.se/research/leimar/fulltext/MultConvStab.pdf

[9] Gandon, S., Jansen, V.A.A., & Van Baalen, M. (2001). Host life history and the evolution of parasite virulence. *Evolution*, 55, 1056-1062.

[10] Van Baalen, M. and Rand, D.A., 1998. The unit of selection in viscous populations and the evolution of altruism. *Journal of theoretical biology*, 193, pp.631-648.
